# Gut Microbiota Affects Age-Related Plasma Metabolites

**DOI:** 10.3390/microorganisms14030602

**Published:** 2026-03-08

**Authors:** Jayanta K. Das, Chee W. Chia, Qu Tian, Angelina Angelova, Luigi Ferrucci, Toshiko Tanaka

**Affiliations:** 1 Longitudinal Studies Section, Translation Gerontology Branch, National Institute on Aging, National Institutes of Health, Baltimore, MD 21224, USA; jayanta.das@nih.gov (J.K.D.); chiac@grc.nia.nih.gov (C.W.C.); qu.tian@nih.gov (Q.T.); ferruccilu@grc.nia.nih.gov (L.F.); 2 Bioinformatics and Computational Biosciences Branch, National Institute of Allergy and Infectious Diseases, National Institutes of Health, Bethesda, MD 20892, USA; angelina.angelova@nih.gov

**Keywords:** gut-microbiota, plasma metabolite, human, aging

## Abstract

Older age is a well-established risk factor for many chronic diseases, yet the biological mechanisms underlying this increased risk are not fully understood. Both gut microbiome composition and the plasma metabolome change with age and may help explain how aging influences disease susceptibility. In this study, we examined the associations between age-related gut microbiota and metabolomic biomarkers in participants of the Baltimore Longitudinal Study of Aging (BLSA), covering a broad age range (27–98 years; 55% female). At the phylum level, we identified four age-associated phyla: Firmicutes, which was negatively associated with age, and Proteobacteria, Euryarchaeota, and Verrucomicrobia, which were positively associated with age. At the genus level, six genera—*Akkermansia*, *Escherichia*, *Klebsiella*, *Methanobrevibacter*, *Oscillibacter,* and *Ruthenibacterium*—were positively associated with age, whereas *Faecalibacterium* and *Longibaculum* were negatively associated with age. Many of these microbial taxa were found to influence one or more aging-related metabolites, mediating their effects across various metabolite classes, including bile acids, amino acids, triglycerides, cholesteryl esters, and phosphatidylcholines. Notably, three metabolites, Asparagine, Sphingomyelin C26:0, and Dihydroceramide (d18:0/24:1), were associated with a decreased risk of mortality, whereas six metabolites—Glycoursodeoxycholic acid, Triacylglyceride (16:1_34:3), Triacylglyceride (18:0_34:3), Phosphatidylcholine aa C32:1, Phosphatidylcholine aa C32:2, and Cholesteryl ester 16:1—were linked to an increased risk of mortality. This study highlights connections between age-associated gut microbial taxa at both the phylum and genus levels as potential mediators of circulating metabolites that are linked to mortality risk.

## 1. Introduction

The gut microbiome, composed of the bacteria residing in the human gastrointestinal tract, is involved in a variety of diverse functions that are important for physiological wellbeing [1,2]. One of the mechanisms by which the gut microbiome influences the host’s health is through metabolites. Metabolites are small molecules arising from the metabolic processes in both the microbial inhabitants and the host and serve as chemical messengers that regulate and coordinate a multitude of physiological functions within the body [3].

Prior studies have shown that the gut microbiota and the circulating metabolome change with age. These age-related changes may influence health trajectories [4] with aging, including the risk of diseases such as inflammatory bowel disease [5], cardiovascular disease [6,7], metabolic syndrome [8,9], neurodegenerative disorders including Alzheimer’s disease [10,11], and mortality [12,13,14]. Indeed, there is an intricate interplay between the microbiome and metabolites that influences numerous physiological pathways, including nutrient metabolism, energy homeostasis, and immune regulation [15,16,17]. Most studies have examined the association of the gut microbiome or plasma metabolome with age separately rather than simultaneously within the same study. Several studies have aimed to map the link between the circulating metabolome and gut microbiota [18]. These efforts have resulted in an atlas of plasma metabolomic signatures of gut microbiota that allows researchers to explore the association between gut microbiota composition and plasma metabolites. However, the extent to which the gut microbiome–metabolite relationship contributes to aging and age-related outcomes remains unclear. Understanding the impact of age-associated gut microbiota on circulating metabolites may provide critical insights into the pathogenesis of age-related conditions and facilitate the identification of novel biomarkers and therapeutic targets [19,20,21,22,23].

There are several potential mechanisms through which the microbiome may impact plasma metabolism, as previously reviewed [24,25,26]. One prominent pathway involves the production and processing of metabolites by gut microbes, such as short-chain fatty acids (SCFAs) [27], bile acids [28], trimethylamine N-oxide (TMAO) [29], and peptides [30,31]. These microbial metabolites can enter circulation and have systemic effects, including lipid metabolism, glucose homeostasis, and inflammation. Many of these processes are major risk factors for chronic diseases, and the gut microbiota may be a catalyst for the changes that occur with aging. Additionally, the gut microbiota can modulate the absorption and metabolism of dietary nutrients, affecting plasma levels of metabolites like amino acids, lipids, and vitamins [32,33], thus gut microbiota changes with older age could impact the host’s nutritional status. Furthermore, microbial dysbiosis with aging can disrupt gut barrier integrity, leading to increased translocation of microbial products into the circulation, which can trigger immune responses and metabolic disturbances [34].

To identify the plasma metabolites that are impacted by gut microbiota associated with age, we utilized data from the Baltimore Longitudinal Study of Aging to (1) characterize the gut microbiota composition associated with age, (2) identify plasma metabolites whose levels are affected by age-associated gut microbial taxa, and (3) test whether the gut microbiome-mediated plasma metabolites are associated with all-cause mortality. The results from this study shed light on the link between the gut microbiome and aging, demonstrating the impact of age-associated microbial taxa on circulating metabolites and highlighting the microbiome–metabolite axis as a potential target for promoting healthy aging and preventing age-related diseases.

## 2. Materials and Methods

### 2.1. Study Population

The Baltimore Longitudinal Study of Aging (BLSA) is a population-based study of aging that commenced in 1958. This enduring open-cohort study has provided invaluable insights into the aging process. Detailed protocols and comprehensive information regarding the BLSA cohort have been extensively documented [35]. In essence, the BLSA cohort is a rolling enrollment study that includes men and women residing in the Washington DC–Baltimore area, who are continuously monitored at intervals of approximately 2–4 years, tailored to their respective age groups. The assessments were conducted over a 2–3 day stay at the Intramural Research Program of the National Institute on Aging (IRP, NIA) Clinic Research Unit, or via home visits for those with significant debilitation. During a study visit, participants undergo a thorough evaluation of physical, functional, and cognitive assessments. During these visits, demographic information on age, sex, and self-reported race was collected. Self-reported race was grouped into three categories of White, Black, and other. The other category included participants who identified as Chinese, Filipino, Japanese, Hawaiian, other Asian or other Pacific Islander, American Indian or Alaska Native, not classifiable, or other non-white. The present analysis encompasses 704 BLSA participants for whom we have data on plasma metabolomics and the gut microbiome. Ethical oversight was ensured through approval from the Institutional Review Board (IRB) of the National Institutes of Health, with participants providing informed consent at each visit.

### 2.2. Gut Microbiota Profiling

Fecal samples were collected by a trained nurse at the NIA IRP clinic during the study visit and stored at −80 °C. Extraction of DNA for sequencing was conducted at Diversigen (Corebiome; Minneapolis, MN, USA) using Qiagen PowerFecal Pro extraction kit. A DNA library was prepared using the Nextera library preparation kit (Illumina, San Diego, CA, USA), and shallow shotgun sequencing was conducted using the the Diversigen^®^ BoosterShot^®^ (New Brighton, MN, USA) pipeline with the Illumina NextSeq 500 instrument (Illumina, San Diego, USA). DNA sequences were filtered for low quality score (Q-Score <20 for front and <15 for tail sequences) and length (<60 bp) using fastp [36]. Taxonomic decontamination (host removal) and subsequent community profiling were performed with the Kraken2 tool [37] against the human genome (GRCh38.p13) and the reference genomes of bacteria, archaea, viruses, fungi, and protozoa organisms extracted from RefSeq databases (December 2020). The abundance of each operational taxonomic unit (OTU) forming the microbial profiles of each sample was represented by read counts. The OTUs were filtered to remove rare taxa using a cut off of raw count sum ˃0 in at least 1% of samples. As a result, we had 9328 OTUs. For alpha diversity analyses, samples were rarefied to a minimum sequencing depth to account for unequal sequencing depth. After sequence processing, sequencing depth across samples ranged from 28,010 to 22,784,194 reads per sample (median = 4,535,716; mean = 4,762,228). For compositional analysis, raw counts were converted to relative abundances (RA) and CLR (Centered Log-Ratio) transformed [38]. Taxa (both at phylum and genus levels) with prevalence greater than 0.5% across all samples were used for downstream analysis.

### 2.3. Plasma Metabolite Measurements

Overnight fasted plasma samples stored at −80 °C were used to assess plasma metabolites following detailed protocols described elsewhere [39]. In brief, metabolites were extracted and measured using liquid chromatography tandem mass spectrometry (LC–MS/MS) at Biocrates Life Sciences AG (Innsbruck, Austria) using the MxP^®^ Quant 500 kit. Quality control was performed where metabolites with values below the limit of detection (LOD) were set to missing, and metabolites with >30% missing samples were excluded from the analyses. For metabolites with ≤30% missing, values were set at half the minimum value. There were 466 out of 622 metabolites that passed quality control.

### 2.4. Statistical Analysis

All analyses were conducted with RStudio version 2023.06.2 using R version 4.2.2. The ‘phyloseq’ package in R [40] was utilized for the initial data processing analysis, exploration, and visualization of microbiome data. Then transform function in the ‘microbiome’ package in R [41] is used to apply transformations of microbiome count data into relative abundance (RA) and CLR (Centered Log-Ratio) at the phylum and genus levels. Also, the estimate richness function in the ‘microbiome’ package is used to compute alpha diversity metrics from microbiome species count data.

The alpha diversity of our samples was assessed using three commonly used metrics: observed species, the Shannon diversity, and Pielou’s evenness index. The observed species metric quantifies the number of distinct taxa present in each sample, reflecting species richness. The Shannon diversity index incorporates both richness and evenness providing an overall measure of community diversity. Pielou’s evenness index, calculated as the Shannon index divided by the natural logarithm of species richness, specifically measures the uniformity of species distribution and ranges from 0 to 1, with values closer to 1 indicating greater evenness. While the Shannon index provides an overall assessment of diversity, the Pielou index specifically quantifies the uniformity of species distribution within a community, offering valuable insights into the ecological balance of the microbial population.

The associations between microbial taxa (phylum and genus level) and age was evaluated using multiple linear regression, where microbial taxa served as the dependent variable and age served as the independent variable. The models were adjusted for sex and race. Statistical significance was considered at false discovery rate corrected *p*-value (FDRp) ≤ 0.05 unless otherwise specified in the text.

Causal mediation analysis was utilized to uncover the mediating effect of the age-associated microbiome on plasma metabolites using the “mediation” package implemented in R [42]. We estimated the extent to which microbial taxa mediate the relationship between age and plasma metabolites. Significant mediation was defined as an average mediation effect being greater than 20% and the direct effect being no longer significant (*p* > 0.05).

Cox proportional hazards models [43] were utilized to understand the associations between individual metabolites and all-cause mortality. Follow-up time was calculated from the assessment of metabolites (time 1 for BLSA) to the date of death. For those not censored, follow-up time was calculated from the time of metabolite assessment to the last recorded study visit date. The same covariates used in the linear regression models were included: age at assessment of metabolites, sex, race, and technical batch.

## 3. Results

### 3.1. Characteristics of Study Participants

In our study, we analyzed a total of 704 participants, with females constituting 55% (n = 387) and males 45% (n = 317) of the cohort (Table S1). The average age of the participants was 72.05 years (SD ± 11.89), with age ranging from 27 to 98 years, reflecting a predominantly older adult population. Approximately two-thirds of the participants self-identified as White, and 27% identified as Black, with the remaining 6% identifiying with other racial groups.

### 3.2. Microbiota Alpha Diversity Changes with Chronological Age

**Figure 1:**
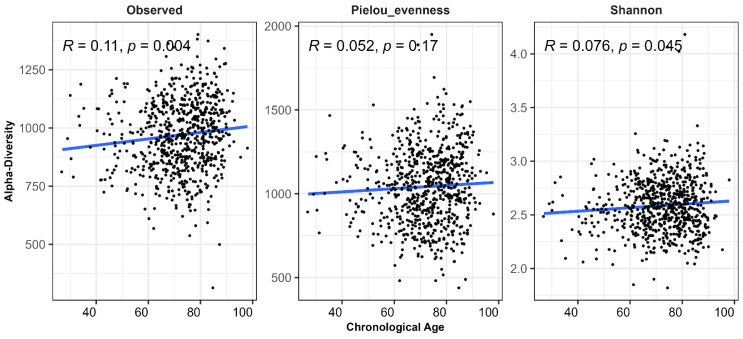
Relationship between alpha diversity and chronological age. Scatter plots with fitted regression lines illustrate the linear associations between chronological age and alpha diversity across three metrics: Observed richness, Shannon diversity, and Pielou’s evenness.

We observed significant associations between age and alpha diversity. Older age correlated with observed species and Shannon diversity (*p* < 0.05 for both; Figure 1), indicating an increase in microbial richness and overall diversity with advancing age. The positive association with observed species suggests that older individuals harbor a greater number of distinct microbial taxa in the gut. Pielou evenness, which reflects the uniformity of species distribution within the microbial community, showed a positive trend with age but did not reach statistical significance (*p* = 0.17). Consistent with these findings, the increase in Shannon diversity—an index that integrates both richness and evenness—appears to be primarily driven by increased species richness, with a contributory trend toward greater evenness.

### 3.3. Association Between Microbial Taxa and Chronological Age

**Figure 2:**
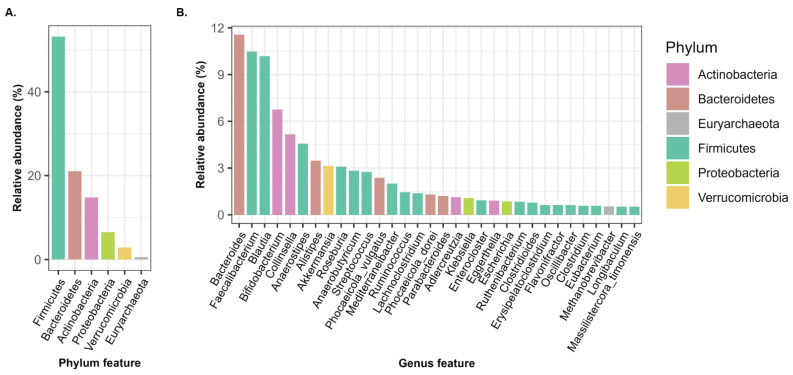
The relative abundances (RA) of the top phylum- and genus-level taxa (RA > 0.5%). (**A**) Bar plot showing the relative abundance (%) of six phyla. (**B**) Bar plot showing thirty-two genera, with colors indicating the corresponding phylum-level classification for each genus.

We conducted a characterization of microbial taxa of BLSA samples at the phylum (a more general taxonomic level) and genus (a more specific taxonomic level) levels. After filtering taxa with relative abundance (RA) ≥ 0.5% across all samples, there were six phylum-level and thirty-two genus-level taxa for subsequent analyses. The most prevalent taxa at the phylum level were Firmicutes (~54%), followed by Bacteroidetes (~21%) and Actinobacteria (~14%), with these three phyla collectively representing over 89% of the total microbial community, indicating their prominence within the gut microbiota (Figure 2A). This distribution is consistent with established literature, where Firmicutes and Bacteroidetes collectively constitute the two predominant phyla, accounting for approximately 90% of the total gut microbiota [44]. At the genus level, we noted *Bacteroides* as the leading taxon, followed by *Faecalibacterium* and *Blautia* (Figure 2B).

**Figure 3:**
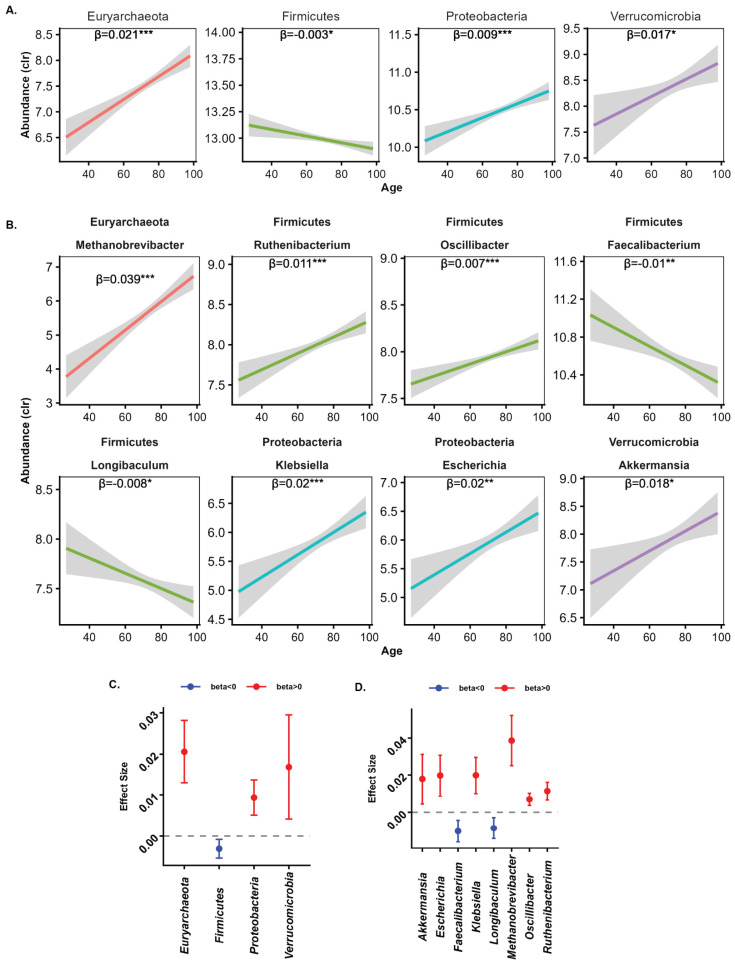
Association between microbial taxa and age. The unadjusted line plot with a fitted linear trend with confidence internvals shows the relationship between microbial phylum-level (**A**) or genus-level (**B**) taxa significantly associated with age. The corresponding forest plots present effect sizes with 95% confidence intervals for the identified phylum (**C**) and genus (**D**) taxa. Statistical significance (adjusted *p*-value) is indicated by asterisks: *** *p* < 0.001, ** 0.001 < *p* < 0.01, * 0.01 < *p* < 0.05.

Among the six phyla examined in the study, four showed a significant association with age (*p*-adj ≤ 0.05; Table S2). Firmicutes were negatively associated with age, while Euryarchaeota, Proteobacteria, and Verrucomicrobia had positive associations with age (Figure 3A,C). The largest average effect sizes were observed for Euryarchaeota, followed by Verrucomicrobia, Proteobacteria, then Firmicutes. Among the 32 genera examined, we identified eight that were significantly associated with age, all belonging to four age-associated phyla: one from Euryarchaeota, four from Firmicutes, one from Proteobacteria, and one from Verrucomicrobia (*p*-adj < 0.05) (Figure 3B,D). Out of the eight genera, six demonstrated a positive correlation with age: *Akkermansia*, *Escherichia*, *Klebsiella*, *Methanobrevibacter*, *Oscillibacter,* and *Ruthenibacterium*. In contrast, the remaining two genera—*Faecalibacterium* and *Longibaculum* (both from the phylum Firmicutes)—exhibited a negative correlation with age. Notably, four of these age-associated genera belong to the phylum Firmicutes, highlighting that taxa within the same phylum display differing associations with age reflecting the complexity of the gut microbiome composition.

### 3.4. Characterization of Plasma Metabolites Mediated by Age-Associated Microbial Taxa

To understand the downstream consequences of age-associated differences in gut microbiota composition, we conducted a mediation analysis to identify plasma metabolites that are mediated by the age-associated gut microbiome. This implies that changes in microbial composition with age may lead to alterations in metabolite abundance, which in turn could impact various physiological processes or health outcomes associated with aging.

**Figure 4:**
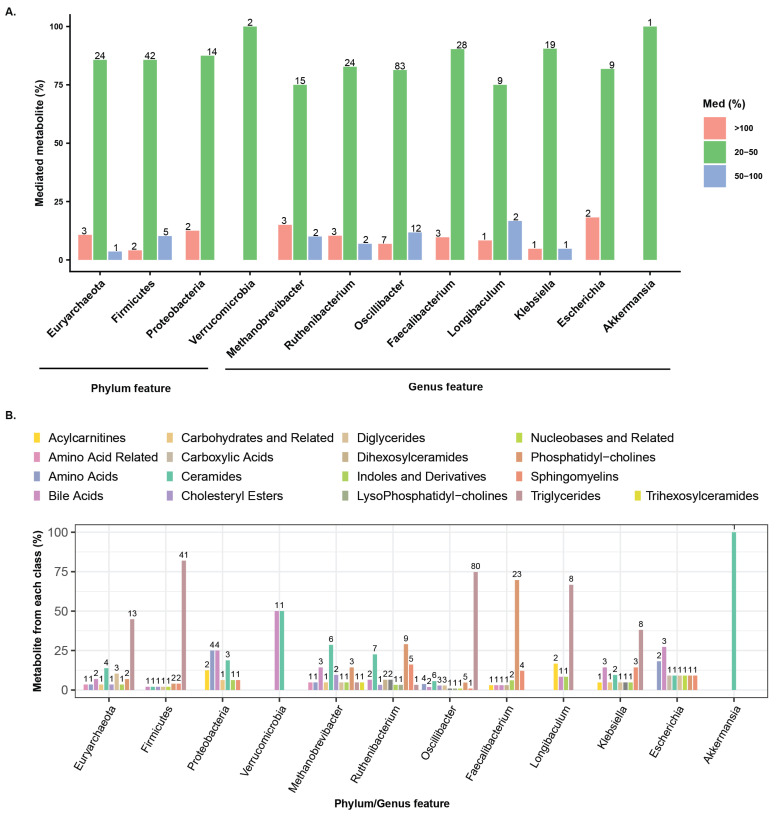
Quantitative analyses highlight age-associated microbial taxa at both the phylum and genus levels and their mediated metabolites. (**A**) Bar plots show the percentage of mediated metabolites (y-axis) for each phylum- and genus-level taxon, grouped into three categories: ≥100%, 51–100%, and 20–50% mediation. Numbers above the bars indicate the exact counts of mediated metabolites. (**B**) Bar plots display the total proportion (%) of mediated metabolites for each metabolite class, with numbers above the bars indicating the exact counts of metabolites in each class.

We analyzed microbial taxa with significant mediation effects, defined by an average causal mediation effect (ACME) *p* < 0.05 and mediation ≥ 20% linking metabolites with age (Table S3, Figure 4A). Most effects ranged between 20–50%, though some exceeded 100%, and a few surpassed 500%. Extremely high values can arise when direct and indirect effects are large but act in opposite directions, causing strong inconsistent mediation. To minimize bias, taxa with mediation effects above 500% were excluded from further analysis. The four age-associated phyla mediated the abundances of 95 metabolites, while eight genera mediated 227 metabolites. Among the phyla, Firmicutes had the largest impact, mediating 49 metabolites across eight classes, including Triglycerides (40 metabolites), Phosphatidylcholines (2), Sphingomyelins (2), and one each from Bile Acids, Ceramides, Cholesteryl Esters, Diglycerides, and Nucleobases and Related (Figure 4B, left). Euryarchaeota mediated ten metabolite classes, including Triglycerides (13), Ceramides (4), Diglycerides (3), Bile Acids (2), and one each from Amino Acid Related, Amino Acids, Carbohydrates and Related, Cholesteryl Esters, Indoles and Derivatives, and Phosphatidylcholines. Proteobacteria primarily influenced seven metabolite classes, including Amino Acids (4), Bile Acids (4), Ceramides (3), and Acylcarnitines (2). Verrucomicrobia had a more limited effect, mediating Bile Acids (1) and Ceramides (1). Among the dominant genera taxa, *Oscillibacter*, *Faecalibacterium*, and *Ruthenibacterium* stood out, mediating 102, 31, and 29 metabolites, respectively (Figure 4B, right). *Oscillibacter* (Firmicutes) mediated the largest number of metabolites, predominantly Triglycerides (76), along with Ceramides, Phosphatidylcholines, Amino Acids, and several other classes. *Faecalibacterium* (Firmicutes) primarily mediated Phosphatidylcholines (21) and Sphingomyelins (4), while *Ruthenibacterium* (Firmicutes) impacted Phosphatidylcholines, Ceramides, Sphingomyelins, and additional classes. *Klebsiella* (Proteobacteria) mediated 21 metabolites, mainly Triglycerides and Bile Acids, and *Methanobrevibacter* (Euryarchaeota) mediated 20 metabolites, notably Ceramides and Bile Acids. Smaller contributions were observed for *Longibaculum* (Firmicutes, 12), *Escherichia* (Proteobacteria, 11), and *Akkermansia* (Verrucomicrobia, 1), reflecting a more limited impact on metabolite mediation. Among the significant metabolites, approximately 36% were commonly mediated by both phyla and genera, while 8% and 56% were uniquely mediated by phyla and genera, respectively (Table S3). This highlights the importance of taxa-level mediation. Furthermore, when comparing the metabolites mediated independently at the phylum and genus levels, many were found to target the same metabolite classes.

**Figure 5:**
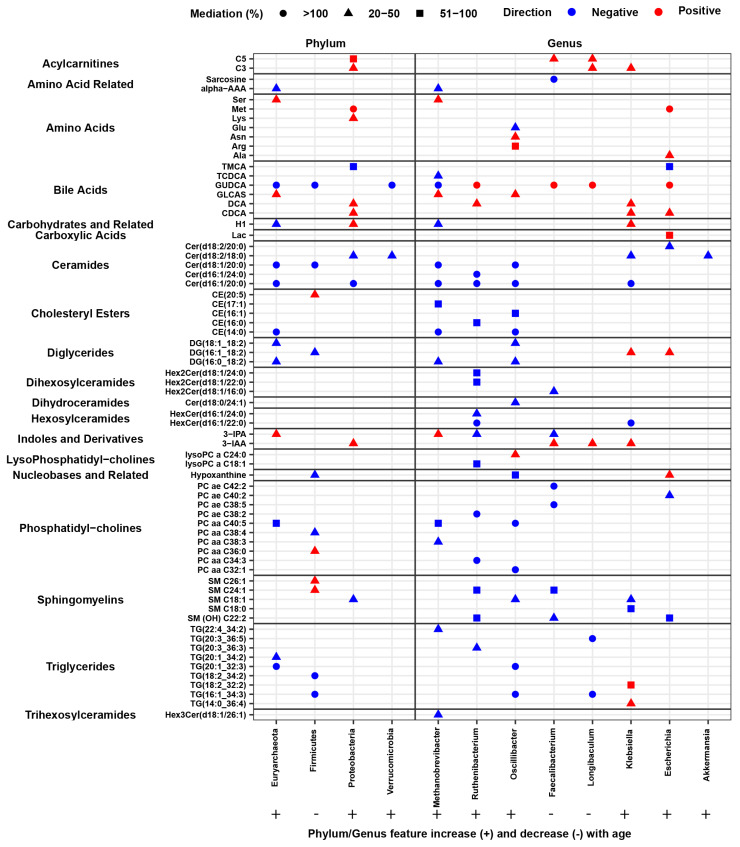
The figure presents the top two metabolites selected based on the highest mediation percentages, illustrating metabolites mediated in both positive and negative directions. Three shapes are used to denote mediation categories: circles represent mediation percentages ≥100%, triangles represent 20–50% mediation, and squares represent 51–100% mediation. The direction of the mediation effect is indicated by color: red denotes a positive average causal mediation effect, while blue denotes a negative average causal mediation effect.

Among the microbial taxa examined, we observed varied impacts on specific metabolites, with some taxa mediating two or more metabolites. Mediation effects (ACME) were both positive and negative, and certain metabolites were influenced in both directions by different microbial taxa. For illustrative purposes, we highlighted the top two metabolites within each class for each microbial taxon exhibiting the highest mediation effect (%), demonstrating how microbial taxa can affect metabolites in both directions (Table S3, Figure 5). At the phylum level, ACME values were exclusively positive or negative, whereas at the genus level both positive and negative mediation effects were observed. At the phylum level, Firmicutes, which is negatively associated with age as discussed, also exhibited negative (inhibitory) mediation effects on several metabolites. In contrast, Euryarchaeota, Proteobacteria, and Verrucomicrobia are positively associated with age. Euryarchaeota and Verrucomicrobia showed positive mediation effects on one metabolite each, whereas Proteobacteria demonstrated a positive mediation effect specifically on Asparagine. At the genus level, as described earlier, two genera are negatively associated with age, while six were positively associated. These genera exhibit complex interactions with metabolites, mediating two or more metabolites across different classes with both positive and negative effects (Table S3, Figure 5).

### 3.5. Association of Microbial Mediated Metabolites with All-Cause Mortality

**Figure 6:**
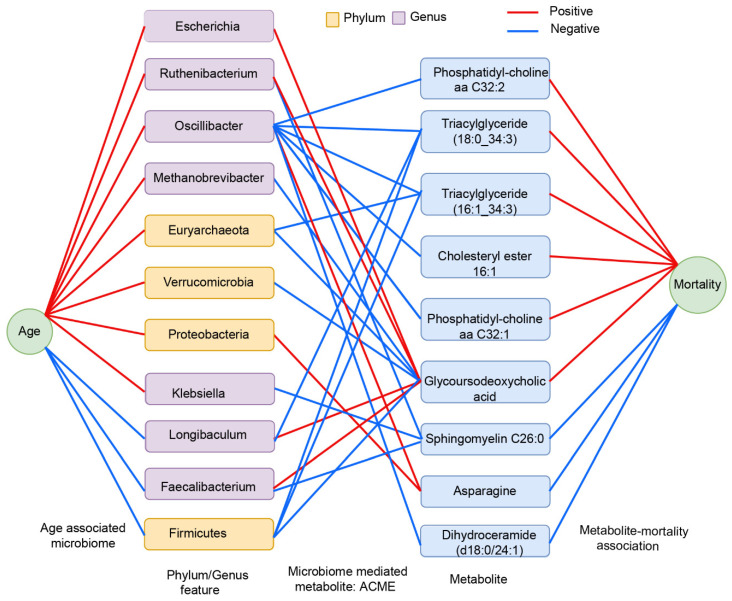
This figure represents age-associated microbial taxa influencing plasma metabolites that are linked to all-cause mortality. It provides a graphical representation of microbial taxa at the phylum and genus levels significantly associated with chronological age and their mediation of metabolites based on significant average causal mediation effects (ACME). The final linkage connects these mediated plasm metabolites to those significantly associated with all-cause mortality. Associations are represented by edges, with red indicating positive associations and blue indicating negative associations.

We then tested the association of metabolites mediated by the age-associated gut microbial taxa with all-cause mortality in 704 participants with data on vital status. There were 92 deaths over an average follow-up period of 3.37 years (up to 9.91 years). Of the 189 metabolites that were significantly mediated by the gut microbiome, nine metabolites were associated with mortality. Three metabolites (Asparagine, Sphingomyelin C26:0, and Dihydroceramide (d18:0/24:1)) were associated with a decreased risk of mortality and six metabolites (Glycoursodeoxycholic acid, Triacylglyceride (16:1_34:3), Triacylglyceride (18:0_34:3), Phosphatidyl-choline aa C32:1, Phosphatidyl-choline aa C32:2, and Cholesteryl ester 16:1) with an increased risk of mortality (Table S4, Figure 6).

## 4. Discussion

The current analysis in a healthy cohort of older adults reveals a complex relationship between age, gut microbiota, and metabolites that reflect both patterns of resilience and decline. The measures of diversity and composition of the gut microbiota are consistent with prior reports in older adults but also reflect patterns that have been reported in centenarians and long-lived individuals [45,46,47]. Using all-cause mortality as an example, we explored whether differences in gut microbial taxa abundance with aging may contribute to life span through the regulation of circulating metabolites. We make several key observations: first, the impact of the age-associated gut microbiome on plasma metabolites is heterogeneous, some having associations with many metabolites while others have a more targeted impact on fewer metabolites. Second, in a healthy population, there is a mix of microbiota–metabolite associations that can be interpreted as detrimental (from the perspective of life span) or protective. By leveraging data on gut microbiota and plasma metabolites, we begin to uncover the impact of the aging gut microbiota and its impact on life span through the regulation of plasma metabolites.

In BLSA participants, microbiome diversity increases with chronological age. Our results are consistent with prior studies that report higher alpha diversity in older adults [48,49]. Studies have shown that gut microbiota diversity changes throughout the lifespan, with the steepest increases observed from birth to adolescence and relative stability during early adulthood and an increase again in older age [49]. The BLSA participants in this report were older, thus our results of increased alpha diversity at older ages are consistent with prior reports [48,49,50,51]. In particular, higher diversity is observed in the oldest old, including centenarians and nonagenarians, suggesting that higher diversity may be a marker of resilience in those who are long-lived [46,48]. A more diverse microbiome is often considered beneficial, as it has been associated with better metabolic functions, enhanced immune responses, and overall better gut health [52]. Consistent with this, studies have found that in people with poorer health at older age, such as those with frailty, have lower alpha diversity compared to healthy controls [46,48,53,]. These results are consistent with those observed in the BLSA cohort, which comprises a generally healthy population where participants must be free of major chronic diseases and have no cognitive or physical disabilities upon enrollment.

The age-associated microbial phyla in the BLSA revealed that Firmicutes were the most abundant phylum observed, and its abundance declined with older age. Like with alpha diversity measures, this age-associated composition difference observed in Firmicutes is consistent with reports in long-lived individuals and centenarians [46,48]. However, there is some conflicting evidence in the literature on the association between Firmicutes and age: while some studies have shown that the abundance of Firmicutes decreases with age, particularly in the oldest old or long lived [50,54], other studies report that the abundance remains stable or increases with age [55,56]. In our study cohort, where participants were on average in their eighth decade, Firmicutes abundance was negatively associated with age. This trend may be attributed to the relatively higher proportion of middle-aged and older participants in our study sample. However, it’s important to recognize that the relationship between Firmicutes abundance and age is multifaceted. Factors such as diet, lifestyle, health status, and geographic location can significantly influence this relationship, highlighting the complexity of microbial dynamics in the context of aging.

We observed an increase with age of several taxa in the BLSA, many of which align with findings from prior studies. For instance, Proteobacteria [49,,57,58], Euryarchaeota [59], and Verrucomicrobia [50,58] have all been reported to be found in higher abundance with older age. These taxa have been associated with key aging outcomes such as cognitive function in older adults. For example, Verrucomicrobia has been linked to better performance in tasks related to psychomotor processing speed, cognitive flexibility, and learning [60,61,62]; Proteobacteria were negatively associated with executive function, learning, and memory. This observation underscores the dynamic changes in microbial composition across different phyla with advancing age.

Many of these age-associations with genera identified in the BLSA were consistent with previous reports. For example, the genera *Escherichia* [57], *Ruthenibacterium* [63], and species *Klebsiella pneumoniae* [64] under the genus *Klebsiella* were observed to increase with age, while the RA of the genus *Faecalibacterium* [65] showed a decrease with chronological age or was lower in older compared to younger adults. Interestingly, while the overall impact of Firmicutes at the phylum level may be negatively associated with age, the direction of age-association at the genus level within Firmicutes was mixed. These differing patterns within Firmicutes likely reflect functional heterogeneity among genera, as distinct taxa may differ in metabolic capacity, ecological niche, and interactions with the host, thereby contributing differently to age-associated physiological changes.

The goal of the current study was to describe the impact of age-associated gut microbiota composition on plasma metabolites as potential mediators of health effects in aging. We observed that Euryarchaeota (increase with age) and Firmicutes (decrease with age) were mediated by the greatest number of metabolites, including bile acids, carboxylic acids, carbohydrates, and various lipid metabolites. *Euryarchaeota* and the genus *Methanobrevibacter,* found in higher abundance with older age, mediated the abundance of conjugated the secondary bile acids Glycoursodeoxycholic acid (GUDCA) and Glycolithocholic acid sulfate (GLCAS). Bile acids are produced in the liver from cholesterol, stored in the gall bladder, and released into the gut in response to feeding. Primary bile acids that reach the colon are processed by the gut microbiota to secondary bile acids and go through various biochemical transformations [66]. One of the microbiome-driven reactions of bile acids is de-conjugation by bile salt hydrolases (BSH). Metagenomic analysis of the gut microbiota shows *Methanobrevibacter* species show BSH activity, which supports the role of this taxa in bile acid metabolism [67]. Interestingly, metagenomic analysis shows that *Firmicutes* has the highest proportion of bacterial clones that display BSH activity. This is consistent with our data, where Firmicutes at the phyla level and four taxa at the genus levels significantly mediated the level of three bile acids (GUDCA, DCA and GLCAS). The impact on bile acid metabolism had important consequences on aging. In the BLSA, GUDCA was positively associated with all-cause mortality. The association linking GUDCA with mortality in older adults is a novel discovery; however, it is consistent with prior work that links changes in bile acids such as lithocholic acid with healthy aging and mortality in humans [68]. Further, prior studies have reported GUDCA was associated with an increased risk of major adverse cardiovascular events [69] and liver cancers [70,71], supporting bile acids playing a role in the lifespan.

Lipid metabolites made up the largest proportion of the targeted assay used in this study; thus, lipids, particularly triglycerides, had the greatest number of associations with gut microbial taxa. The association of gut microbiota with lipid metabolites may be explained in part through their association with bile acids. Bile acids can directly impact circulating lipids through their primary role in aiding absorption of dietary fats, and can further impact lipid metabolism, particularly in the liver. Both primary and secondary bile acids act as ligands and activate nuclear receptors including farnesoid X receptor (FXR). The activation of FXR in the liver can reduce de novo lipogenesis by suppressing SREBP1 expression [72] and promote fatty acid β-oxidation through upregulation of PPARa [73]. In this study, Euryarchaeota was associated with both bile acids, triglycerides, and cholesterol. Similarly, Firmicutes, Proteobacteria, and Verrucomicrobia were mediated by the abundance of both bile acid(s) and lipids. The variability in plasma lipids, that may be partially driven by gut microbiota, has important implications for the lifespan. In our study, two triglyceride metabolites (Triacylglyceride (18:0_34:3) or stearic, Triacylglyceride (16:1_34:3) or palmitoleic), cholesterol ester (16:1), and two Phosphatidylcholines (phosphatidyl-choline (C32:1) and phosphatidyl-choline (C32:1)) were positively associated with mortality, while sphingomyelin (C26:0) was negatively associated with mortality. Both palmitoleic acid (16:1) and stearic acid (18:0) fatty acids have been identified as risk factors for mortality [74,75], potentially through their association with lipoprotein concentrations. Plasma concentrations of sphingomyelins with long chain fatty acids (C20 or greater) have been associated with a reduced risk of mortality [76,77]. Plasma phosphatidylcholine concentration is associated with type 2 diabetes mellitus [78]. While the mechanisms underlying these associations are unclear, sphingomyelins are important modulators of several biological processes including apoptosis [79] and inflammation [80].

Firmicutes and Proteobacteria at the phylum level and the Firmicutes genus *Oscillibacter* significantly mediated plasma abundance of the amino acid asparagine. This is consistent with prior reports from the Swedish CArdioPulmonary bioImage Study, where the majority of species within the Firmicutes phylum showed negative associations with plasma asparagine concentrations [18]. There are endogenous and exogenous factors that contribute to plasma asparagine concentrations including diet and genetics [81,82]. This study provides evidence that the gut microbiota can be one of the contributors to the regulation of circulating concentrations of asparagine. Asparagine in turn showed a negative association with all-cause mortality. Other population studies have described the protective associations between asparagine and type 2 diabetes, cardiovascular disease [83], and mortality [84]. Asparagine has been shown to increase insulin sensitivity [85] and modulation of energy production by serving as an intermediate substrate for the Krebs cycle by serving as an intermediate substrate during cardiac ischemia [86]. Our study provides evidence that differences in abundance of Firmicutes and Proteobacteria may influence plasma asparagine levels, potentially affecting insulin sensitivity and energy utilization, with implications for lifespan.

Our study revealed several important relationships between the gut microbiota and plasma metabolites as they related to all-cause mortality. However, there are several limitations of the study that need to be addressed. First, the analysis of the gut microbiota and plasma metabolites was cross-sectional, thus we cannot make conclusions regarding causality of the associations we observed. We hypothesized that gut microbiota mediates the associations between age and metabolites, however, we cannot rule out the possibility that age-associated differences in plasma metabolites influence gut microbiota composition. For instance, GUDCA has been shown to regulate hepatic production of bile acids and further alters gut microbiota composition both in humans [87] and mice [88]. To further understand the direction of association, future studies should leverage longitudinal study designs or experimental data to understand the temporal relationship between aging, changes in gut microbiota, and changes in plasma metabolites. Second, we used a targeted plasma metabolite panel, which had good coverage of different metabolite classes, but it is likely that we did not measure all gut microbiota-related metabolites in this study. Third, although the BLSA is a well-defined cohort with extensive phenotype data, its participants belong to a high socioeconomic group, which tends to be healthier than the general population. Thus, it is important to evaluate the generalizability of the study findings using data from studies that with diverse demographics. Finally, the current analysis did not include an independent replication, thus validation in other studies is warranted.

In summary, our work contributes to the growing effort to understand the complex relationship between the gut microbiota and plasma metabolites as potential mediators of health effects. By focusing on age-associated gut microbial taxa, we begin to disentangle their effect on the circulating metabolome in aging. We observed mediating effects on secondary bile acids, lipids, and amino acids. Further work is needed to determine whether the age-associated gut microbial taxa can be therapeutic targets to extend lifespan.

## Data Availability

The original contributions presented in this study are included in the article/Supplementary Material. All data from the Baltimore Longitudinal Study of Aging (BLSA) can be accessed through request via proposal submission through the BLSA website (blsa.nih.gov).

## Supplementary Materials

The following supporting information can be downloaded at: https://www.mdpi.com/article/10.3390/microorganisms14030602/s1. Table S1: Study populations and quantitative information. Table S2: Age associated microbiome Phylum feature (A) and Genus feature (B) adjusted for Sex, Race. Estimate (Beta) and significant (*p*-value) are provided. Table S3: Microbiome feature (Phylum feature (A) and Genus feature (B)) and associated mediation metabolite. For each mediated metabolite, metabolite class, ACME Estimate (Beta) and ACME (*p*-Value) are provided. Table S4: List of metabolites associated with mortality risk.
